# VSD可有效管理手术切口感染

**DOI:** 10.3779/j.issn.1009-3419.2018.04.27

**Published:** 2018-04-20

**Authors:** 远阳 赖, 小龙 闫

**Affiliations:** 空军军医大学附属唐都医院胸腔外科 Department of Thoracic Surgery, Tangdu Hospital Affiliated to the Military Medical University of the Air Force

随手术部位感染是第二大健康相关感染，仅次于呼吸道感染。不论是发达国家或是发展中国家，手术切口感染均显著影响患者预后、加重疾病负担，轻者延长住院时间，重者可能甚至需要重症监护治疗甚至危及生命。普胸外科手术切口感染发生率约为3.2%-3.66%^[1, 2]^，肺切除手术后切口感染发生率稍低。切口感染常见原因包括手术切口闭合（缝合）不良、伤口内存留异物以及患者相关因素等^[3]^。相关研究^[1, 2]^探讨了普胸外科手术部位感染（包括切口感染、肺炎、脓胸等）的危险因素，提示男性患者、糖尿病、术前激素使用史、诱导放/化疗史、未预防性使用抗生素、手术类型及手术时间均与感染相关。由此可见，手术切口感染涉及了整个围手术期的诸多因素，其预防有一定复杂性。然而，如同其他健康相关感染，手术切口感染大部分情况下是可以避免的。

2016年，世界卫生组织发布了新版《Global Guidelines For The Prevention Of Surgical Site Infection》^[4]^，推荐术前、术中、术后三阶段共26方面建议，以最大限度预防手术部位感染，减轻手术部位感染所致疾病负担；Lancet Infect Dis基于指南基础上，针对术前^[5]^、术中及术后^[6]^分别细化了13条、16条推荐。证据显示^[7, 8]^，指南的有效实施有利于管理控制手术部位感染，包括切口感染。我国早在2010年就已发布《外科手术部位感染预防和控制技术指南（试行）》^[9]^，该指南基于我国临床实践具体实际，为广大医务工作者临床实践提供了指导，对我国手术部位感染的预防也起着关键性作用。然而，国内指南内容相对简单、部分缺乏循证证据，且时间较为久远、些许观念不乏过时；因此笔者认为，可将世卫组织《Global Guidelines For The Prevention Of Surgical Site Infection》与我国《外科手术部位感染预防和控制技术指南（试行）》结合，以期更好指导临床实践、加强围手术期管理，最大限度降低手术部位感染发生率，改善患者预后、减轻疾病负担。

胸外科手术后切口感染并无红、肿、热、痛等典型炎症反应表现，主要症状体征为体温、血象升高，局部肿胀、压痛，引流后可见胸壁肌肉坏死、脓腔形成。传统处理措施包括充分引流、彻底清创、加强换药、依据药敏试验结果全身使用抗生素及二期缝合等，如马继龙等研究论文所述；但传统治疗时间较长、患者痛苦、心理负担加重。如能快速、有效治疗切口感染，将对降低感染相关死亡率、提高患者术后生活质量、改善预后大有裨益。马继龙等所作研究将广泛用于骨科、整形烧伤科的负压封闭引流装置（VSD）应用于胸外科，对具体应用步骤作了详实描述总结，取得了较好的预期效果，为胸外科手术后切口感染的处置另辟了一条安全、有效、可行的蹊径，有较大的推广意义。研究结合了胸外科特殊性与VSD基本作用原理，分析了VSD应用于胸外科手术切口感染的理论先进性和实际可行性，论据充分，论证合理。此外，文章还强调了VSD应用过程中的关注点及细节，对进一步推广应用有较大的启示作用。虽然该研究有较好的初步结论，但笔者认为，在未来仍需较大样本量的、定量清晰的对照试验，以提供更多循证医学证据，提高论证强度；此外，该研究仅归纳总结了Ⅲ类切口术后切口感染的病例，进一步研究可适当扩大纳入标准，以期研究更加全面与完善。
